# Severe Fever with Thrombocytopenia Syndrome, Japan, 2013–2017

**DOI:** 10.3201/eid2604.191011

**Published:** 2020-04

**Authors:** Yusuke Kobayashi, Hirofumi Kato, Takuya Yamagishi, Tomoe Shimada, Tamano Matsui, Tomoki Yoshikawa, Takeshi Kurosu, Masayuki Shimojima, Shigeru Morikawa, Hideki Hasegawa, Masayuki Saijo, Kazunori Oishi, SFTS Epidemiological Research Group Japan

**Affiliations:** National Institute of Infectious Diseases, Tokyo, Japan

**Keywords:** severe fever with thrombocytopenia syndrome virus, severe fever with thrombocytopenia syndrome, SFTS, SFTSV, Phenuiviridae, epidemiology, companion animal, viruses, tickborne diseases, vector-borne infections, zoonoses, Japan, Huaiyangshan banyangvirus

## Abstract

We conducted an epidemiologic study of severe fever with thrombocytopenia syndrome (SFTS) in Japan during 2013–2017. Of 303 cases reported during that period, 133 (44%) were included in this study. The median time between onset of illness and diagnosis of SFTS shortened, from 11.5 to 3.0 days, but the case-fatality rate remained high, at 27%. In 64 patients (48%), a close contact with companion animals was reported within 2 weeks of disease onset. Of these 64 patients, 40 were surveyed further, and we confirmed that 3 had direct contact with body fluids of ill companion animals; 2 had direct contact with the saliva of an ill feral cat or pet dog. These patients reported no history of tick bite, suggesting that ill companion animals might be a source of SFTS virus transmission. Direct contact with the body fluids of ill companion animals should be avoided.

Severe fever with thrombocytopenia syndrome (SFTS) is an emerging tickborne infectious disease, identified in 2009 in the rural areas of Hubei and Henan provinces in China (*1*,*2*); a total of 7,419 cases were reported from 23 provinces during 2010–2016 (*3*). SFTS is endemic not only to China but also to South Korea and Japan (*4*,*5*). Huaiyangshan banyangvirus (formerly SFTS virus [SFTSV]), the causative agent of SFTS, belongs to the genus *Banyangvirus* in the family *Phenuiviridae*. Although the name of the virus has recently been changed from SFTSV to Huaiyangshan banyangvirus by the International Committee on Taxonomy of Viruses (*6*), the term SFTSV is still used. SFTSV is found in tick species such as *Haemaphysalis longicornis*, *Amblyomma testudinarium*, and *Ixodes nipponesis* in China, South Korea, and Japan (*7*–*9*). Antibodies to SFTSV were detected in wild and domestic animals, such as goats, deer, cattle, dogs, and cats, in SFTS-endemic areas of these 3 countries (*10*–*15*). SFTSV is thought to circulate in an enzootic environment and to have a tick–vertebrate–tick cycle (*12*). In addition, human-to-human transmission through blood and respiratory secretions has been reported from China and South Korea (*16*–*18*).

Since the first case of SFTS was reported in Japan in 2013 (*5*), ad hoc retrospective and prospective surveillance has been conducted by Japan’s Ministry of Health, Labor, and Welfare. A total of 23 suspected SFTS cases were retrospectively reported from 2005, of which 11 were confirmed through this surveillance (*5*,*19*). SFTS was included in Japan’s Infectious Diseases Control Law as a category IV notifiable disease on March 4, 2013. Our previous report describing the nationwide epidemiology of SFTS during April 2013–September 2014 demonstrated that the case-fatality rate (CFR) in 49 patients was as high as 31% (*20*). As of October 2017, a total of 303 confirmed cases had been reported to Japan’s National Epidemiologic Surveillance of Infectious Disease (NESID) (*21*).

The annual numbers of SFTS patients reported each year during 2013–2017 were 40, 61, 60, 59, and 83, respectively. These patients were reported from 23 of the 47 prefectures in Japan, and the geographic distribution of the SFTS cases expanded gradually each year from western to central Japan (Appendix Figure 1). Most patients had SFTS onset during April–October (Appendix Figure 2), and the annual national notification rates of SFTS ranged from 0.03 to 0.06 cases/100,000 person-years over the study period. These findings were similar to those previously reported for 2013 and 2014 (*20*).

Studies conducted in China, South Korea, and Japan reported that several clinical and laboratory parameters were associated with a fatal outcome of SFTS (*20*,*22*–*25*). Because no effective therapeutic agents for SFTS are currently available (*22*,*25*,*26*), effective and specific treatments for SFTS must be developed. A recent case report demonstrated that a veterinarian who cared for 3 symptomatic cats, 2 of which were pets, was infected with SFTSV (*27*). On the basis of such information, we conducted questionnaire surveys of SFTS patients to collect information regarding their direct contact with ill companion animals 2 weeks before illness onset. We conducted a retrospective observational study to identify the changes in the epidemiologic findings of SFTS patients over the study period of March 2013–October 2017 to determine the prognostic factors for SFTS and to evaluate the possible risk of direct exposure to ill companion animals possibly infected with SFTSV.

## Methods

### National Surveillance of SFTS in Japan

NESID defines a case of SFTS as illness in a patient with fever or gastrointestinal symptoms, any laboratory findings, including thrombocytopenia (<10.0 × 10^4^/μL), leukopenia (<4,000 cells/μL), or elevated liver enzymes, plus laboratory confirmation of SFTSV infection. The confirmatory examinations include detection of the SFTSV genome using reverse transcription PCR (RT-PCR), which was performed at the local public health institute, the National Institute of Infectious Diseases (Tokyo, Japan), or both, and SFTSV-specific antibody testing using an immunofluorescence assay or an indirect immunoperoxidase assay (*13*,*28*,*29*).

### Study Design and Data Collection

We performed a retrospective observational study of SFTS cases reported to NESID during March 2013–October 2017. Physicians were asked to participate in this study by completing a questionnaire sent by mail. Demographic data, social history of outdoor activity for 2 weeks before illness onset, clinical symptoms, and laboratory data for SFTS patients were collected through the first questionnaire. The physicians who agreed to participate in this study collected the information about the patients through their medical charts or by telephone interviews with patients or their family members, after obtaining informed consent. We collected clinical information and laboratory data for the acute phase (within 2 weeks after illness onset). We obtained data for patients whose cases were reported during March–September 2014 from a previous study (*18*); our study added data collected during October 2014–October 2017. We extracted basic demographic data for each SFTS patient reported during the study period from the NESID database.

We sent a second questionnaire to the physicians who responded to the first questionnaire; the second questionnaire requested information about whether the confirmed SFTS patients had contact with companion animals (such as cats and dogs). The information requested included whether the SFTS patient had close contact with an ill companion animal within 2 weeks before illness onset or direct contact with body fluids of an ill companion animal and the outcome for the ill companion animal.

### Statistical Analyses

We used SPSS Statistics 21.0 for Windows (IBM, https://www.ibm.com) for all statistical analyses. We performed trend analysis for different years by using the Spearman rank correlation test and the Jonckheere–Terpstra test. We used the Pearson χ^2^ test or the Wilcoxon rank-sum test to compare the characteristics of survivors and nonsurvivors. We used logistic regression modeling for multivariable analysis. All p values were 2-sided, and p<0.05 was considered statistically significant.

### Ethics Approval

This study was conducted under approval by the Medical Research Ethics Committee of the National Institute of Infectious Diseases (approval no. 706). All aspects of the study complied with the Helsinki Declaration. Each patient or their proxy provided written informed consent.

## Results

### Epidemiologic and Clinical Characteristics of Study Cases

**Figure 1:**
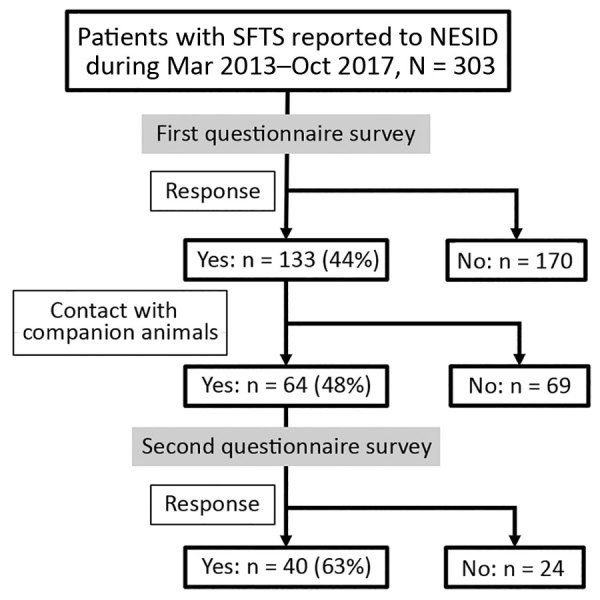
Flow diagram of epidemiologic study of 133 patients with severe fever with thrombocytopenia syndrome, Japan, March 2013–October 2017. NESID, National Epidemiologic Surveillance of Infectious Disease; SFTS, severe fever with thrombocytopenia syndrome.

Of 303 patients reported to NESID, we included 133 (43.9%) whose demographic and clinical data were collected through the first questionnaire in this study (Figure 1). Of these 133 patients, 130 (98%) had SFTS diagnosed by detection of an SFTSV-specific genome using RT-PCR and 3 (2%) by detection of an SFTSV-specific antibody. We observed no significant difference in sex and age distribution between these 133 patients and the 170 patients for whom no questionnaire was collected. The median ages of the 2 groups were 73 years (interquartile range [IQR] 65–82 years) for the patients with questionnaires and 75 years (IQR 67–82 years) for those without. 

**Figure 2:**
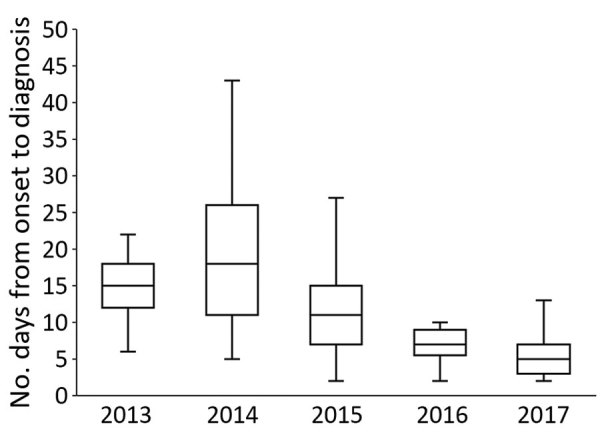
Comparison of time between illness onset and confirmed diagnosis in 133 patients with severe fever with thrombocytopenia syndrome, Japan, March 2013–October 2017. We conducted a trend analysis of time from initial visit to diagnosis over the study period by using the Jonkheere–Trapstra test (p<0.01). In the box plots, the bottom boundary of the box indicates the 25th percentile, the line within the box marks the median, and the top boundary of the box indicates the 75th percentile. Whiskers above and below the box indicate the 10th and 90th percentiles.

**Figure 3:**
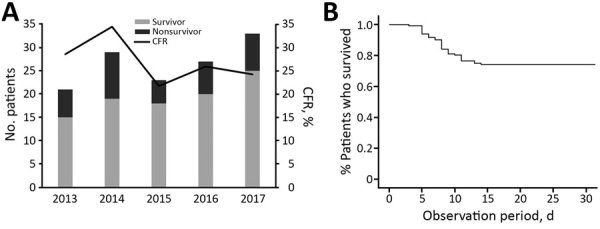
Annual number and CFRs and Kaplan–Meier survival curve of 133 patients with severe fever with thrombocytopenia syndrome, Japan, March 2013–October 2017. A) Trend analysis of CFRs over the study period by using Spearman rank correlation test (p = 0.285). B) Kaplan-Meier curve of 133 patients with severe fever with thrombocytopenia syndrome within 30 days after illness onset. CFR, case-fatality rate.

Although the median time from illness onset to initial hospital visit was 3 days (IQR 2–5 days) and did not differ significantly between years, the median time between illness onset and a confirmed diagnosis for 133 SFTS cases shortened significantly over the study period (p<0.01) (Figure 2). The median time between illness onset and a confirmed diagnosis also significantly shortened over the study period for 97 survivors and 36 nonsurvivors (p<0.01). Thirty-six patients died, corresponding to a CFR of 27% (Figure 3, panel A). We observed no significant difference in age or sex distribution by year between the 97 survivors and 36 nonsurvivors. The Kaplan–Meier survival curve of 133 SFTS patients demonstrated that most deaths (94%) occurred within 2 weeks after illness onset (Figure 3, panel B).

Underlying illnesses among these 133 patients included hypertension (n = 47), diabetes mellitus (n = 27), and dyslipidemia (n = 15), as well as a few instances of malignant disease (n = 9) (Table 1). The proportion of patients with malignant disease was significantly higher among the nonsurvivors (17%) than among the survivors (3%) (p<0.05). Sixty-four (48%) patients were reported to have been bitten by a tick, and 55 (41%) had traces of a tick bite; 109 (82%) had participated in outdoor activities on hills and in fields. Sixty-four (48%) had close contact with companion animals, such as dogs and cats, within 2 weeks before illness onset.

**Table 1:** Demographic characteristics of 133 patients with severe fever with thrombocytopenia syndrome, Japan, March 2013– October 2017*

Characteristic		No. (%)			p value†
		All case-patients, n = 133	Nonsurvivors, n = 36	Survivors, n = 97	
Sex					
M		63 (47)	17 (47)	46 (47)	0.984
F		70 (53)	19 (53)	51 (53)	
Median age, y (IQR)		73 (65–82)	78 (68.25–84.75)	72 (63.5–80)	0.015‡
Underlying conditions					
Malignant tumor		9 (7)	6 (17)	3 (3)	0.006
Diabetes mellitus		27 (20)	7 (19)	12 (12)	0.860
Hypertension		47 (35)	14 (39)	33 (34)	0.707
Dyslipidemia		15 (11)	3 (8)	12 (12)	0.381
None		36 (27)	8 (22)	28 (29)	0.444

*Values are no. (%) unless indicated. IQR, interquartile range. †Pearson χ^2^ test used for all variables except age. ‡Wilcoxon rank-sum test.

We reviewed clinical symptoms and laboratory data at initial hospital visit of the 133 patients with available information (Appendix Tables 1, 2). We found no significant difference in neurologic symptoms, except for tremor, between survivors and nonsurvivors. The serum levels of aspartate aminotransferase, alanine aminotransferase, blood urea nitrogen, lactic acid dehydrogenase, and potassium were significantly higher, and activated partial thromboplastin time significantly longer, in nonsurvivors than in survivors.

### Prognostic Factors

To investigate possible prognostic factors at the initial hospital visit, we performed multivariable analysis by selecting factors on the basis of the univariate analysis results, past reports, and clinical importance (Table 2). The multivariable logistic regression analysis indicated that a low platelet count was associated with a fatal outcome (odds ratio [OR] 1.38, 95% CI 1.07–1.78). The complications of malignant disease (OR 20.83, 95% CI 1.32–327.70) and presence of tremor at initial hospital visit (OR 17.37, 95% CI 1.26–239.39) also were associated with an increased risk for death.

**Table 2:** Multivariable analysis of prognostic factors for 133 patients with severe fever with thrombocytopenia syndrome, Japan, March 2013–October 2017*

Variable	Univariable			Multivariable	
	OR (95% CI)	p value		OR (95% CI)	p value
Age	NA	0.015		1.07 (0.98–1.16)	0.115
Malignant tumor	6.13 (1.45–26.04)	0.006		20.83 (1.32–327.70)	0.031
Disorientation	1.58 (0.71–3.52)	0.259		1.37 (0.30–6.35)	0.687
Tremor	8.60 (1.57–47.04)	0.004		17.37 (1.26–239.39)	0.033
Platelet	NA	0.661		1.38 (1.07–1.78)	0.014
Albumin	NA	0.791		0.99 (0.15–6.62)	0.994
ALT	NA	0.015		1.01 (1.00–1.02)	0.371
LDH	NA	0.012		1.00 (1.00–1.00)	0.378
CK	NA	0.591		1.00 (1.00–1.00)	0.842
APTT	NA	0.007		1.06 (0.97–1.16)	0.215

*ALT, alanine aminotransferase; APTT, activated partial thromboplastin time; CK, creatine kinase; LDH, lactate dehydrogenase; NA, not applicable; OR, odds ratio.

### Contact with Ill Companion Animals

Of 64 patients who responded to the second questionnaire, 40 (62.5%) reported contact with a companion animal within the 2 weeks before illness onset (Figure 1). For 5 of these patients, their companion animal appeared to be ill during this period, and 3 (patients 1, 2, and 3) had direct contact with the body fluids of the ill companion animals (Table 3). These three patients had no history of a tick bite. No information was available about the type of body fluid to which patient 1 was exposed. Patient 2 had direct contact with the saliva of a symptomatic feral cat, and she was also bitten by this cat, which subsequently died. No virologic examination of these 2 cats was attempted. Patient 3 had direct contact with the saliva of an ill dog that he owned. RT-PCR detected the presence of SFTSV genome in blood from this dog.

**Table 3:** Characteristics of 3 patients with severe fever with thrombocytopenia syndrome who had contact with body fluid of an ill companion animal before illness onset, Japan, March 2013–October 2017*

Patient no.	Onset year	Age, y/sex	Outcome	Tick bite	Species of ill companion animal	Direct exposure to ill animal’s body fluid	Outcome of ill companion animals	SFTSV detection from animals
1	2014	46/F	Recovered	No	Cat	Yes	Recovered	Not tested
2	2016	57/F	Died	No	Cat	Yes (bite)	Died	Not tested†
3	2017	42/M	Recovered	No	Dog	Yes	Recovered	Yes

*SFTSV, severe fever with thrombocytopenia virus (Huaiyangshan banyangvirus). †Clinical and laboratory findings were similar to those of human case.

## Discussion

In this study, we investigated the epidemiologic and clinical features of 133 SFTS patients identified in Japan during 2013–2017. The overall CFR was 27%, which did not significantly change over the study period. We found a significant reduction in the interval between illness onset and the SFTS diagnosis and identified that underlying malignant disease, low platelet count, and appearance of tremor at hospital visit were significantly associated with increased risk for death after adjusting for age.

On the basis of national surveillance data, as of October 31, 2017, the proportions of fatal cases during the study period were 35% in 2013, 26% in 2014, 18% in 2015, 14% in 2016, and 13% in 2017 (https://www.niid.go.jp/niid/ja/sfts/sfts-idwrs/7415-sfts-nesid.html). Therefore, a discrepancy was noted between the CFRs observed in this study and the proportions of fatal cases in the national surveillance database. We found that the time between illness onset and hospital visit remained unchanged but that the time between illness onset and diagnosis shortened significantly, indicating an increased awareness of this disease among physicians during more recent years in the SFTS-endemic areas of Japan. Because physicians who diagnose a case of SFTS are requested to report the case to NESID immediately after diagnosis, the time between illness onset and reporting had also been shortened (from 14 days [IQR 11–24 days] during 2013–2014 to 6 days [IQR 4–8 days] in 2017).

Our findings demonstrate that deaths commonly occurred within 2 weeks after illness onset (Figure 3, panel B). During the study period, 11 patients (1 patient each in 2013 and 2015, 2 patients in 2016, and 7 patients in 2017) died after their diagnosis was reported to NESID. This fact might explain, in part, the discrepancy between the CFRs in this study and the proportions of fatal cases in national SFTS surveillance data.

Our finding that a low platelet count at initial hospital visit was a risk factor for a fatal outcome is in agreement with the results of previous studies (*20*,*22*,*23*). Although age-adjusted underlying malignant disease also was significantly associated with fatal outcome in our study, whether it has a direct effect on the survival of SFTS patients remains uncertain because of the limited number of such cases (n = 9). A previous study based on univariable analysis showed that the proportion of patients with tremor at admission was not significantly higher in fatal cases (20%) than in nonfatal cases (3%) (*20*), but our multivariable analysis demonstrated that appearance of tremor at initial hospital visit was significantly associated with a fatal outcome.

Three patients had direct contact with the body fluid of ill companion animals (1 dog and 2 cats) before illness onset, and these patients reported no history of tick bite. Importantly, 2 of the 3 patients had direct contact with the saliva of an ill feral cat or an ill pet dog. In addition, the cat that bit patient no. 2 subsequently died (Table 3). These findings suggested that the ill companion animals could be the direct source of SFTSV infection.

Since 2017, a total of 24 pet cats living in western Japan have been diagnosed with SFTSV infection (*30*). All cats infected with SFTSV showed acute onset of clinical signs. High fever (>39.5°C) was noted in 15/22 cats (68.2%) and vomiting in 10/24 cats (41.7). Animal experiments by Park et al. (*31*) confirmed that cats are susceptible to SFTSV infection; the authors reported that 4/6 cats infected with SFTSV died, and all cats showed signs that were similar to or more severe than those signs observed in humans infected with SFTSV. Those authors also found high viral loads in serum, saliva, and eye swabs taken 7 days postinfection from cats that subsequently died from infection. Collectively, these findings suggest that SFTSV transmission in patient no. 2 (Table 3) very possibly occurred from an ill cat through the patient’s direct contact with the saliva of the cat.

Our study has several limitations. First, only 133/303 (43.9%) patients reported to NESID were included in the study. Although we found no significant difference in sex and age distribution between the 133 patients who participated in this study and the 170 patients who did not, the 133 study participants might not represent the population of interest for research purposes. Second, although 3 patients who had contact with a body fluid of ill companion animals reported no history of a tick bite, whether these patients actually had a tick bite that was not reported because of recall bias is unknown. Therefore, we cannot exclude the possibility that the animals carried ticks and that a tick bite, rather than exposure to body fluid, might have been the actual mode of transmission. Third, no information is available on whether the 3 SFTS patients possibly infected from their ill companion animals were infected with the same strain of SFTSV as the animals. For any patients possibly infected from their companion animals, testing should be performed to determine whether the viruses isolated from the patient and from their animal are identical. In our study, we conducted the questionnaire surveys to evaluate the SFTSV infections from companion animals but were unable to determine the risk for SFTSV infection from these companion animals. Therefore, further case–control studies are required to determine the risk of exposure to the body fluids of companion animals.

In conclusion, we demonstrated that underlying malignant disease, low platelet count, and appearance of tremor at the first hospital visit were significantly associated with a fatal outcome among SFTS patients. The CFR of SFTS patients in Japan remained high at 27%. Three (2%) of 133 SFTS patients had direct contact with the body fluids of ill companion animals but no reported history of tick bite within the 2 weeks before illness onset, suggesting that ill companion animals might be a source of SFTSV transmission to humans. Although further studies on the epidemiologic and virologic analyses are needed, the owners of companion animals and veterinarians in the SFTS-endemic area should be fully aware of the risk of direct contact with body fluids of ill companion animals. The owners should avoid direct contact with body fluids, such as saliva, of their ill companion animals and should take care not to be bitten by them.

Appendix Additional information regarding severe fever with thrombocytopenia syndrome, Japan, 2013–2017.
