# Supplementary material for: Severe Fever with Thrombocytopenia Syndrome, Japan, 2013–2017
Source: Emerg Infect Dis. 2020 Apr;26(4):692–9. doi: 10.3201/eid2604.191011 (PMC7101122; doi:10.3201/eid2604.191011)
Supplement: Appendix — Additional information regarding severe fever with thrombocytopenia syndrome, Japan, 2013–2017. [file 19-1011-Techapp-s1.pdf]

Article DOI: https://doi.org/10.3201/eid2604.191011

Severe Fever with Thrombocytopenia Syndrome, Japan, 2013–2017

Appendix

Appendix Table 1. Clinical features of SFTS patients at the time of hospital visit in Japan, March 2013–October 2017

	No. (%)			
Symptoms or signs	All patients, N = 133	Nonsurvivors, n = 36	Survivors, n = 97	p-value*
Fever	109 (82)	32 (89)	77 (79)	0.334
Gastrointestinal symptoms	113 (85)	31 (86)	82 (85)	0.799
Vomiting	26 (20)	7 (19)	19 (20)	0.891
Diarrhea	57 (43)	17 (47)	40 (41)	0.541
Abdominal pain	23 (17)	5 (14)	18 (19)	0.668
Lymphadenopathy	28 (21)	7 (19)	21 (22)	0.974
Arthralgia	11 (8)	3 (8)	11 (11)	0.901
Myalgia	18 (14)	5 (14)	13 (13)	0.691
Headache	20 (15)	6 (17)	14 (14)	0.514
Cough	4 (3)	1 (3)	3 (3)	0.96
Sore throat	7 (5)	2 (6)	5 (5)	0.833
Fatigue	87 (65)	27 (75)	60 (62)	0.058
Neurologic symptoms	80 (60)	22 (61)	58 (60)	0.877
Disturbance of consciousness	43 (32)	15 (42)	28 (29)	0.259
Dysarthria	7 (5)	2 (6)	5 (5)	0.918
Convulsion	3 (2)	2 (6)	1 (1)	0.118
Weakness	20 (15)	4 (11)	16 (16)	0.707
Tremor	7 (5)	5 (14)	2 (2)	0.004
Bleeding tendency	21 (16)	6 (17)	15 (15)	0.808
Gingival hemorrhage	8 (6)	4 (11)	4 (4)	0.095
Subcutaneous hemorrhage	12 (9)	2 (6)	10 (10)	0.385

*Pearson’s chi-square test

Appendix Table 2. Laboratory data of SFTS patients at the time of hospital visit in Japan, March 2013–October 2017

		Median [IQR]			
Laboratory data	Normal range	All patients, N = 133	Non-survivors, n = 36	Survivors, n = 97	p-value*
Leukocyte (cells/μL)	3,300–8,600	1670 [1275–2535]	1505 [1123–2063]	1700 [1350–2700]	0.129
Hb (g/dL)	M: 13.7–16.8; F: 11.6–14.8	14.0 [12.8–14.9]	13.9 [12.7–15.1]	14.0 [12.9–14.9]	0.804
Platelet ($\times10^4$/μL)	15.8–34.8	6.6 [4.8–9.7]	6.0 [4.1–9.2]	6.6 [5.0–9.9]	0.206
TP (g/dL)	6.6–8.1	6.4 [6.0–6.9]	6.4 [5.9–6.8]	6.4 [6.0–7.0]	0.769
Alb (g/dL)	4.1–5.1	3.6 [3.1–3.9]	3.4 [2.8–3.8]	3.7 [3.3–4.0]	0.06
T-Bil (mg/dL)	0.4–1.5	0.5 [0.4–0.6]	0.6 [0.3–0.7]	0.4 [0.4–0.6]	0.045
AST (IU/L)	13–30	129 [61–267]	227 [96–414]	117 [54–207]	0.004
ALT (IU/L)	M: 10–42; F: 7–23	58 [36–111]	69 [46–181]	53 [29–95]	0.015
LDH (IU/L)	124–222	435 [315–823]	598 [396–956]	416 [298–706]	0.012
ALP (IU/L)	106–322	205 [169–264]	212 [172–353]	202 [167–257]	0.175
BUN (mg/dL)	8–22	20.9 [15.3–28.0]	24.6 [17.1–38.3]	20.0 [14.0–25.7]	0.008
Cr (mg/dL)	M: 0.65–1.07; F: 0.46–0.79	0.90 [0.72–1.15]	1.04 [0.79–1.40]	0.83 [0.70–1.10]	0.05
CRP (mg/dL)	0.00–0.14	0.27 [0.06–0.52]	0.37 [0.08–0.79]	0.18 [0.06–0.50]	0.078
CK (IU/L)	M: 59–248; F: 41–153	326 [149–964]	368 [187–1235]	284 [143–964]	0.591
AMY (IU/L)	44–132	96 [66–133]	121 [70–154]	91 [65–110]	0.074
Na (mEq/L)	138–145	134 [131–137]	134 [132–137]	135 [131–137]	0.936
K (mEq/L)	3.6–4.8	3.9 [3.6–4.2]	4.1 [3.8–4.2]	3.8 [3.5–4.2]	0.021
Cl (mEq/L)	101–108	100 [97–103]	100 [98–103]	100 [96–103]	0.404
APTT (sec)	24.5–33.8	44.9 [38.7–50.9]	47.0 [43.6–56.2]	43.0 [37.3–48.9]	0.007

IQR, interquartile range; leukocyte, leukocyte; Hb, hemoglobin; TP, total protein; Alb, albumin; T-Bil, total bilirubin; AST, aspartate transaminase; ALT, alanine aminotransferase; LDH, lactate dehydrogenase; ALP, alkaline phosphatase; BUN, blood urea nitrogen; Cr, creatinine; CRP, C-reactive protein; CK, creatine kinase; AMY, amylase; Na, sodium; K, potassium; Cl, chlorine; APTT, activated partial thromboplastin time.

*Wilcoxon rank sum test.

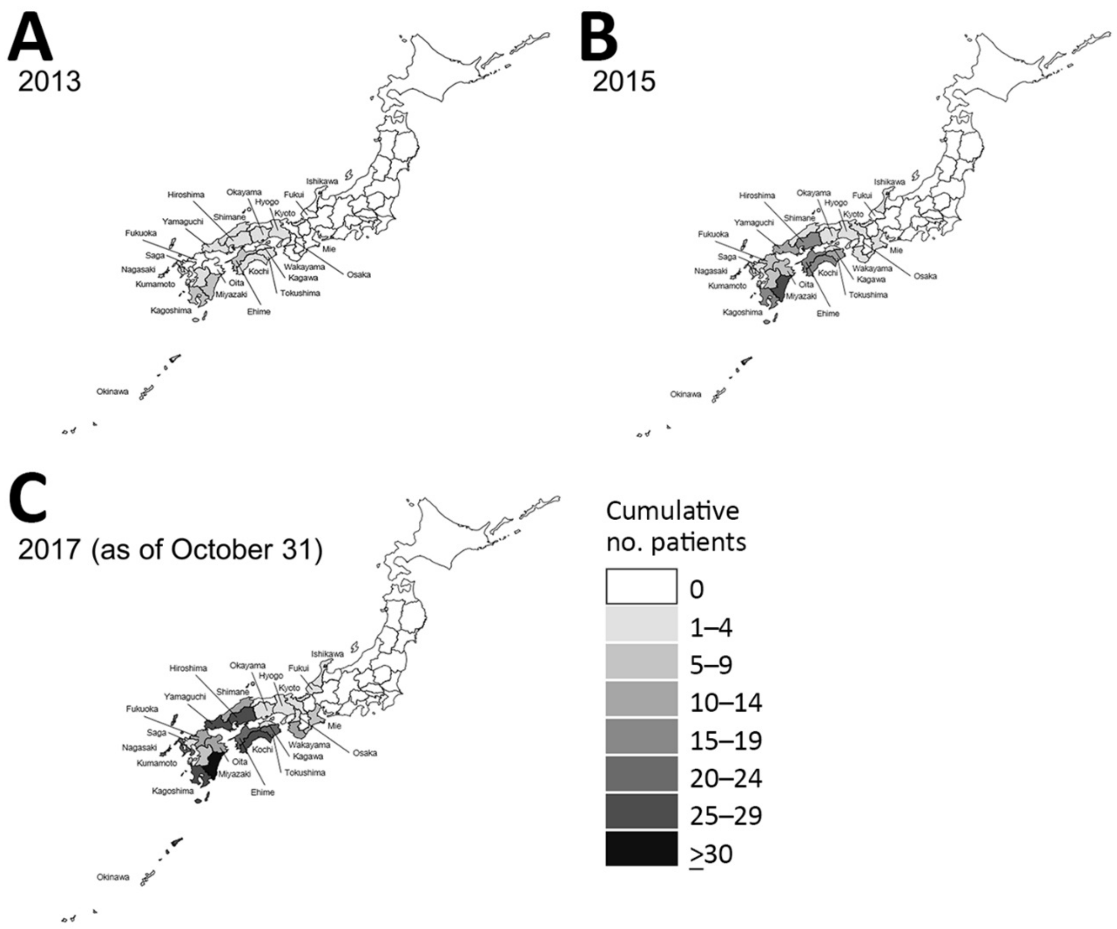

Appendix Figure 1. Geographic distribution of 303 SFTS cases in Japan (cumulative number), March 2013–October 2017. Cumulative number of reported patients by prefecture up to 2013 (A), up to 2015 (B), and up to October 31, 2017 (C).

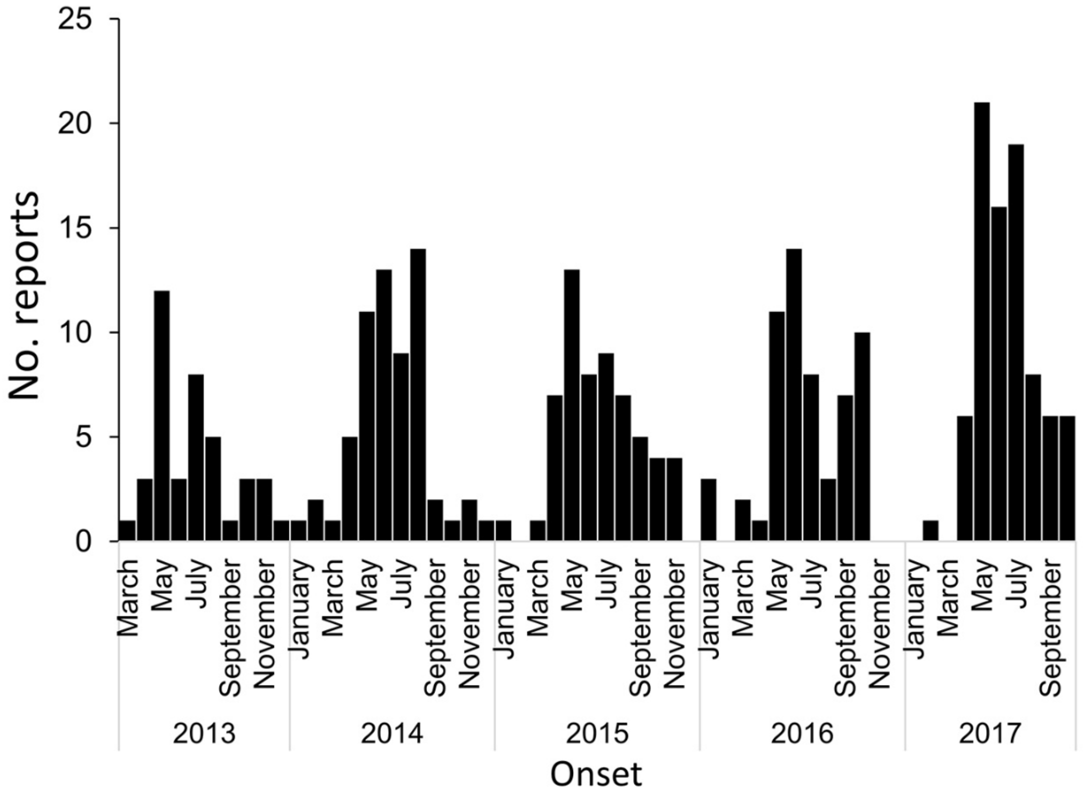

Appendix Figure 2. Seasonal distribution of 303 SFTS cases in Japan, March 2013–October 2017. Gray bars indicate the reported number of SFTS patients by month.
